# Decrease in Circulating Dendritic Cell Precursors in Patients with Peripheral Artery Disease

**DOI:** 10.1155/2015/450957

**Published:** 2015-04-15

**Authors:** Daniel Kretzschmar, Ilonka Rohm, Sebastian Schäller, Stefan Betge, Rudin Pistulli, Yevgeniya Atiskova, Hans-R. Figulla, Atilla Yilmaz

**Affiliations:** Department of Internal Medicine I, Jena University Hospital, Friedrich-Schiller-University Jena, Erlanger Allee 101, 07740 Jena, Germany

## Abstract

Peripheral artery disease (PAD) is a common manifestation of atherosclerosis. Inflammation is important for initiation and progression of the disease. Dendritic cells (DCs) as antigen-presenting cells play an important role in the immune system. Therefore, we hypothesize that, in patients with PAD, DCPs might be reduced in blood due to their recruitment into the vascular wall and induce a proinflammatory response. The numbers of myeloid DCPs, plasmacytoid DCPs, and total DCPs were analyzed by flow cytometry in blood of patients with PAD (*n* = 52) compared to controls (*n* = 60). Femoralis plaques (*n* = 12) of patients who underwent surgery were immunostained for CD209 and CD83 (mDCs) as well as CD304, CD123 (pDCs), and HLA-DR. In patients with PAD, a significant decrease in mDCPs, pDCPs, and tDCPs was observed. In immunostaining, markers indicative for mDCs (CD209: 16 versus 8 cells/0.1 mm^2^, *P* = 0.02; CD83: 19 versus 5 cells/0.1 mm^2^, *P* = 0.03) were significantly elevated in femoralis plaques compared to control vessels. We show for the first time that mDCPs, pDCPs, and tDCPs are significantly reduced in patients with PAD. Immunohistochemical analysis unraveled that the decrease in DCPs might be due to their recruitment into atherosclerotic plaques.

## 1. Introduction

Peripheral artery disease (PAD) was firstly described in 1846 by Brodie [1] and is one of the most common manifestations of atherosclerosis. Approximately 27 million individuals in the industrialized countries are affected by PAD [2]. The presence of PAD is an independent predictor of cardiac and cerebral ischemic events [3–6], and individuals with PAD are at high mortality risk [7, 8]. Among the three major atherosclerotic manifestations, PAD has the highest proatherosclerotic risk profile [9].

Inflammatory processes play a major role in the development of atherosclerosis [10, 11], and are important for the initiation and progression of PAD. Many studies demonstrate a close link between inflammation and PAD. Correspondingly, in apparently healthy men, the relative risk to develop PAD significantly increases with each quartile of baseline C-reactive protein (CRP) concentration [12]. In the Edingburgh Artery Study, inflammatory molecules like CRP, interleukin-6, lipoprotein (a), and soluble intercellular adhesion molecule-1 were found to be connected with increased PAD risk [13]. One study unraveled a proinflammatory phenotype of monocytes and myeloid dendritic cells (mDCs) in patients with PAD [14].

Dendritic cells (DCs), firstly described in 1868 by Paul Langerhans, are potent antigen-presenting cells which have been shown to be present in nearly all tissues [15]. Several subtypes of DCs have been described, the so-called myeloid DCs, which are important to drive an immune answer against bacterial and fungi infections, and plasmacytoid DCs, which are necessary to fight against viral infections. With myeloid DCs and plasmacytoid DCs being predominant, DCs play an important role in the pathogenesis of various inflammatory diseases. As link between the innate and the adaptive arm of the immune system, they patrol the blood in order to initiate an immune response against foreign antigens [16]. DCs are recruited from the blood into peripheral tissues where they are present in an immature state. Upon antigen contact, immature DCs undergo a maturation process in lymph nodes and subsequently migrate to target organs, where they stimulate the cellular immune response via activation of T-lymphocytes through upregulation of antigen-presenting molecules (MHC classes I and II), adhesion molecules (CD11a,b; CD50; CD54; CD58), and costimulatory molecules (CD40; CD80; CD86). In 1995, the presence of DCs was firstly detected in the arterial wall, and it was suggested that they might be involved in the inflammatory pathogenesis of atherosclerosis [17]. In several studies, a significant decrease in circulating dendritic cell precursors (DCPs) was reported in patients with coronary artery disease (CAD) and acute myocardial infarction [18–21]. This decrease is thought to be the result of an enhanced recruitment of circulating DCPs into the atheromata in CAD or the infarcted tissue. In patients with cerebrovascular disease, DCs could be found in carotid plaques [22].

Beyond this work demonstrating the role of DCPs in CAD and patients with cerebrovascular disease [18, 19, 21], the knowledge of their function and role in PAD is very limited [14]. Patients with PAD are exposed a high cardiovascular mortality [23]. The aim of our present study was to analyze the number of circulating mDCPs and pDCPs in patients with PAD in comparison to healthy individuals.

## 2. Methods

### 2.1. Patients and Controls

In our present study, we enrolled patients with PAD who were admitted to the Department of Internal Medicine I of Jena University Hospital. These patients suffered from intermittent claudication (IC), rest pain (RP), or critical limb ischemia (CLI) with peripheral ulceration and had a reduced (<0.8) ankle brachial index (ABI) and suspicious ultrasound findings. IC, RP, and CLI were defined according to the Rutherford (RF) Classification. All patients were screened for coronary artery disease by receiving stress test and transthoracal echocardiography. Those with suspicious findings underwent coronary angiography first. Patients with diseases that could interact with the analysis, such as infections, cancer, autoimmune diseases, hyperthyroidism, and renal insufficiency [24], and medication with immunosuppressive agents were excluded. To exclude measurement interactions with the interventions (PTA), follow-up measurements were done when patients were resubmitted or referred to our outpatient department at least 4 weeks after initial presentation. Routine blood analyses were immediately analyzed after blood withdrawal according to clinical standards.

The patients were divided into the following three subgroups: (1) the control group (*n* = 60) which consisted of subjects with no history of CAD or PAD (normal ABI and cardiac stress test as well as normal transthoracic echocardiogram), (2) patients with typical IC, RP, or CLI (*n* = 52) (no evidence of existing CAD), and (3) patients with PAD and CAD (*n* = 14).

The study was performed in accordance with the Declaration of Helsinki (2000) and was approved by the local ethics committee. Each participant gave informed written consent.

### 2.2. Analysis of Circulating Dendritic Cell Precursors (DCPs) by Flow Cytometry

Circulating mDCPs and pDCPs were analysed by four-colour staining and FACS analysis in fresh blood samples collected in tubes containing EDTA using the Blood Dendritic Cell Enumeration kit (BDCA kit, Miltenyi Biotec). Circulating mDCPs and pDCPs were identified according to their expression of BDCA-1 and BDCA-2 and the absence of the expression of other peripheral blood mononuclear cell (PBMC) markers (CD14, CD 19). For this purpose, 300 *μ*L of blood was mixed with 20 *μ*L of the control cocktail for isotype control and 300 *μ*L of blood was mixed with 20 *μ*L of the anti-BDCA cocktail for cell staining. To discriminate dead cells, 10 *μ*L of a fluorescent cell-impermeant dye was added and samples were incubated for 10 min under 60 W light bulb. The red blood cell lysis solution was used in order to remove erythrocytes. Subsequently, cells were washed and fixed using fix solution. Another solution was added to the samples for optimal dead cell discrimination even after prolonged storage (Miltenyi Biotec).

**Figure 1:**
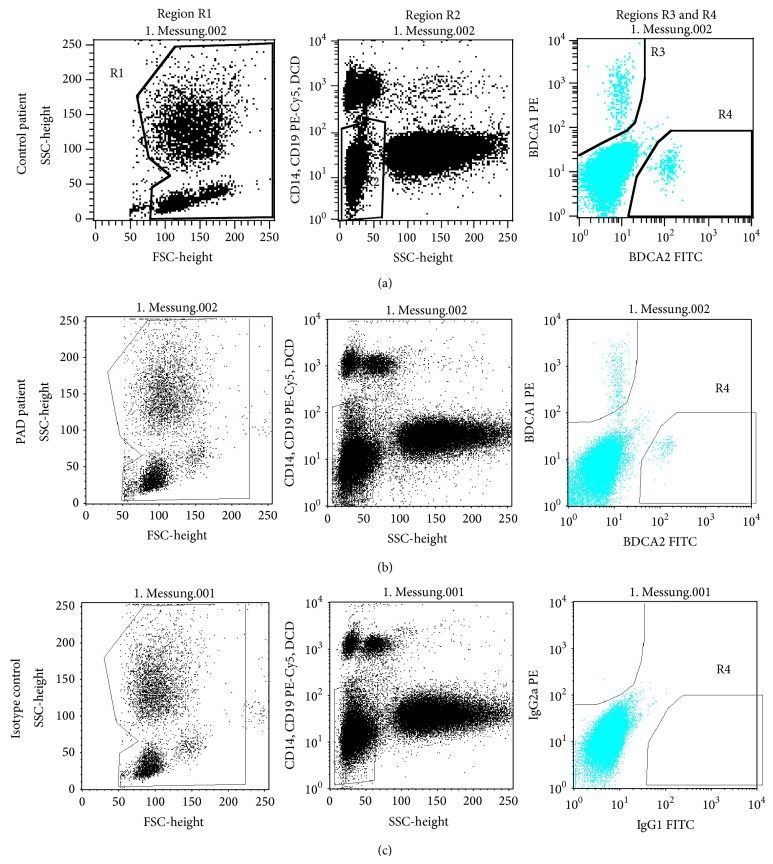
Gating strategy used for the identification of circulating mDCPs, pDCPs, and tDCPs by flow cytometry. (a) DCP analysis in control patients; (b) DCP analysis in patients with PAD; (c) isotype control. Region R1: white blood cells (WBCs) were separated from debris and platelets using their forward and side scatter (FSC and SSC). Region R2 was used to exclude granulocytes by SSC, B lymphocytes by CD19 staining, monocytes by CD14 staining, and dead cells by propidium iodide-staining. In regions R3 and R4, circulating mDCP and pDCP were detected according to their specific BDCA-1 and BDCA-2 staining, respectively.

FACS analysis was performed using FACSCalibur flow cytometer with CellQuest software (Becton Dickinson). As circulating DCPs comprise only 0.1–1% of white blood cells (WBCs), a special gating strategy was used to analyze the number of mDCPs, pDCPs, and tDCPs (Figure 1). In region R1, 2 ∗ 10^5^ white blood cells (WBCs) were registered defined by forward scatter (FSC) and side scatter (SSC), region R2 was used to exclude granulocytes according to their high SSC, and lymphocytes, monocytes, and dead cells were excluded according to their CD14, CD19, and propidium iodide staining, respectively. Circulating mDCPs and pDCPs were detected in regions R3 and R4 due to their specific staining for BDCA-1 and BDCA-2, respectively. Total DCPs (tDCPs) were defined as the sum of cells in regions R3 and R4.

The relative cells numbers of circulating DCPs were assessed as percentage of WBCs. In addition, the absolute cells numbers (cells/*μ*L) were calculated by multiplying the relative cells numbers with the individual WBC count, which was measured in routine laboratory in the same blood sample.

### 2.3. Immunohistochemical Analysis

Plaque specimens of 12 patients undergoing endarterectomy of femoral arteries were immunhistochemically analyzed. Indications for femoral endarterectomy were high grade stenosis <3–10 cm in length causing relevant reductions in the patients walking distance [23]. Duplex scanning, MR imaging, or angiography was performed prior to surgery for quantification of the degree of stenosis. As control tissue, 9 arterial vessels of suicide or accident victims that did not show any macroscopical or microscopical signs of atherosclerosis were used.

Plaque specimens were fixed in 4% formalin and areas with plaque formation were cut out for IHC analysis. Totally occluded vessels were excluded from the study. Plaques used for the present study were at an advanced stage (types IV to VI) according to the AHA classification [25]. After 4-week decalcification in EDTA, plaques were embedded in paraffin. Serial sections (4 *μ*m) were cut and mounted on glass slides. Trichrome staining was performed to visualize the different plaque regions (lipid core, fibrous cap, plaque shoulders, media, and contralateral wall) that were analyzed for the occurrence of DCs as described before [26].

Immunohistochemical analysis of DCs was performed with the antibodies listed in Table 1. Catalyzed signal amplification technique (CSA System, DakoCytomation, Hamburg, Germany) was used according to manufacturers' instructions. Irrelevant isotype-matched antibodies served as appropriate negative controls.

Digital images of the different plaque regions were taken with a CCD-camera (Zeiss AxioCam HRC, Jena, Germany) at a magnification of 100x. Cells were digitally counted in random areas (0.1 mm^2^) in each plaque region using digital image processing software (Axiovision, Zeiss, Jena, Germany). For each quantification, the colour threshold for immunostained cells was manually adjusted until the computerized detection matched the visual interpretation. The mean cells number for each plaque was calculated from the cells numbers seen in the different plaque regions. An intra- and interobserver variability for the results of less than 10% was detected.

### 2.4. Statistical Analysis

Data are given as median or mean. *P* < 0.05 was considered to be statistically significant. For normal distribution, statistical analysis was performed by Student's *t*-test, and for nonparametrically distributed data the Mann-Whitney Rank Sum test was used. Categorical clinical data (e.g., atherogenic risk factors, medication, and gender) were compared between the study groups using chi-square test or Fisher's exact test depending on sample size. For statistical analysis, Prism V4.0b statistical software package was used.

## 3. Results

### 3.1. Baseline Characteristics

Baseline characteristics of patients with PAD and control individuals are reported in Table 2. In our study, 52 patients with PAD (and no evidence of existing CAD) were compared with 60 control patients. Patients with PAD were more often male and had more often the cardiovascular risk factors: smoking and hyperlipidemia. On the other side, total cholesterol and LDL cholesterol were significantly higher in the control patients due to the fact that patients with PAD received more often statins. Leukocyte counts were comparable between the two groups whereas CRP was higher in the PAD group. As expected, the ABI index was significantly (*P* < 0.0001) lower in the PAD group (0.3) compared to 1.0 in the control patients. In addition, we included 14 patients with PAD and CAD; the clinical data of these patients were comparable to the patients with PAD only (data not shown).

### 3.2. Decrease in Circulating mDCPs and pDCPs in PAD

**Figure 2:**
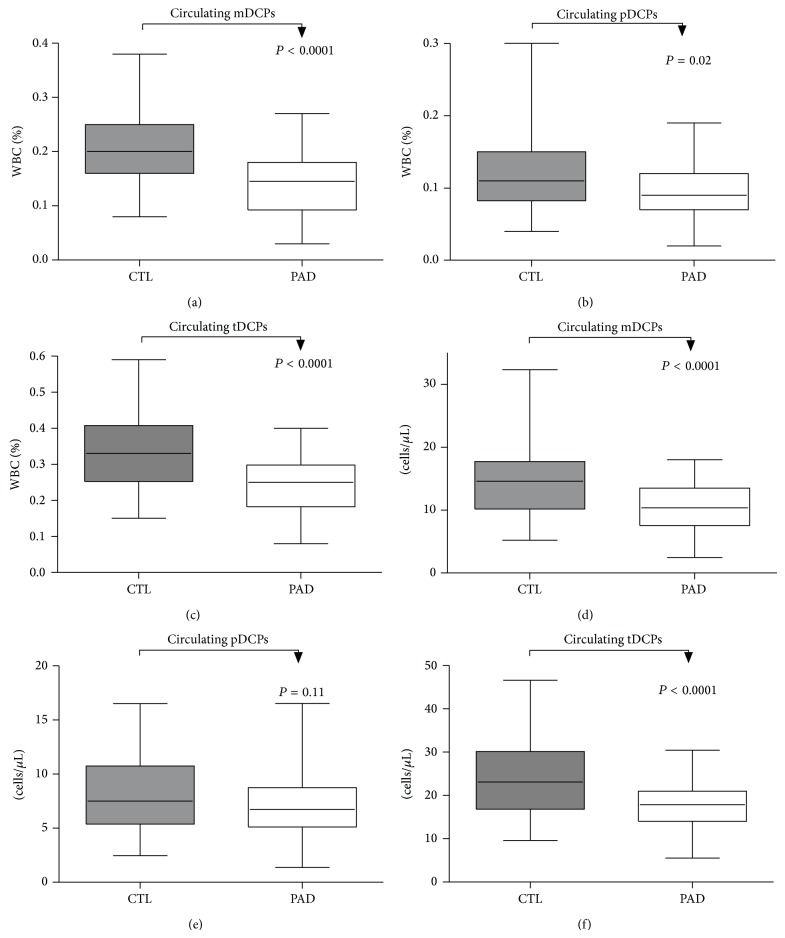
Frequency of circulating mDCPs, pDCPs, and tDCPs in patients with PAD compared with healthy controls. Relative numbers of circulating mDCPs (a), pDCPs (b), and tDCPs (c) shown as percentage of white blood cell count (% of WBCs) and absolute numbers of circulating mDCPs (d), pDCPs (e), and tDCPs (f) (cells/*μ*L) are shown. The box plots indicate the median (line inside the box), 25th and 75th percentile (lower and upper boundary of the box), and 10th and 90th percentile (whiskers outside box).

In patients with PAD, a highly significant decrease in the relative numbers of circulating mDCPs (0.15 versus 0.2; *P* < 0.0001), pDCPs (0.09 versus 0.12; *P* = 0.01), and tDCPs (0.25 versus 0.36; *P* < 0.0001) was observed compared to the controls (Figure 2). Total numbers of mDCPs (10.38 versus 15.00 cells/*μ*L; *P* < 0.0001), pDCPs (6.72 versus 8.40 cells/*μ*L; *P* = 0.03), and tDCPs (17.84 versus 24.54 cells/*μ*L; *P* < 0.0001) were also significantly decreased in patients with PAD.

As long as relative as well as absolute numbers were reduced in patients with PAD, the possibility that the decrease was caused by a dilution of DCPs by an increase in another PBMC population could be excluded.

### 3.3. Decrease in Circulating DCPs according to Rutherford Classification

According to the Rutherford classification, there were 12 patients with Rutherford II, 25 patients with Rutherford III, 6 patients with Rutherford IV, and 9 patients with Rutherford V. We found no significant intergroup differences for circulating DCPs for relative numbers (mDCPs RF II: 0.13; RF III: 0.15; RF IV: 0.16; RF V: 0.14; *P* = n.s./pDCPs RF II: 0.08; RF III: 0.09; RF IV: 0.12; RF V: 0.09; *P* = n.s./tDCPs RF II: 0.26; RF III: 0.25; RF IV: 0.27; RF V: 0.26; *P* = n.s.) as well as for absolute numbers (mDCPs RF II: 9.58; RF III: 10.80; RF IV: 11.46; RF V: 10.56 cells/*μ*L; *P* = n.s./pDCPs RF II: 6.31; RF III: 6.24; RF IV: 9.08; RF V: 7.52 cells/*μ*L; *P* = n.s./tDCPs RF II: 16.96; RF III: 17.92; RF IV: 21.40; RF V: 19.04 cells/*μ*L; *P* = n.s.).

### 3.4. Follow-Up Measurement

**Figure 3:**
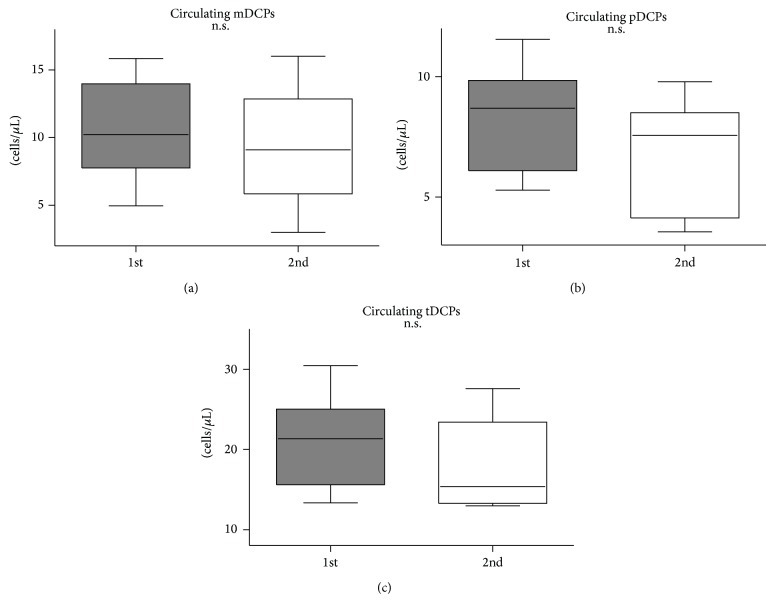
Follow-up frequency of circulating mDCPs, pDCPs, and tDCPs in patients with PAD compared with initial measurement. Absolute numbers of circulating mDCPs (a), pDCPs (b), and tDCPs (c) (cells/*μ*L) are shown. The box plots indicate the median (line inside the box), 25th and 75th percentile (lower and upper boundary of the box), and 10th and 90th percentile (whiskers outside box).

In the 8 patients who attended the follow-up measurement, DCPs were measured at least 4 weeks after initial DCP determination. For these 8 patients with follow-up measurements, we found comparable levels of relative and absolute numbers of mDCPs (0.16 versus 0.14 and 10.23 versus 9.10 cells/*μ*L; *P* = n.s.), pDCPs (0.11 versus 0.07 and 8.96 versus 7.62 cells/*μ*L; *P* = n.s.), and tDCPs (0.29 versus 0.25 and 21.33 versus 15.37 cells/*μ*L; *P* = n.s.), compared to the 2nd measurement (Figure 3).

### 3.5. Comparison of Circulating DCPs in Patients with PAD and PAD/CAD

**Figure 4:**
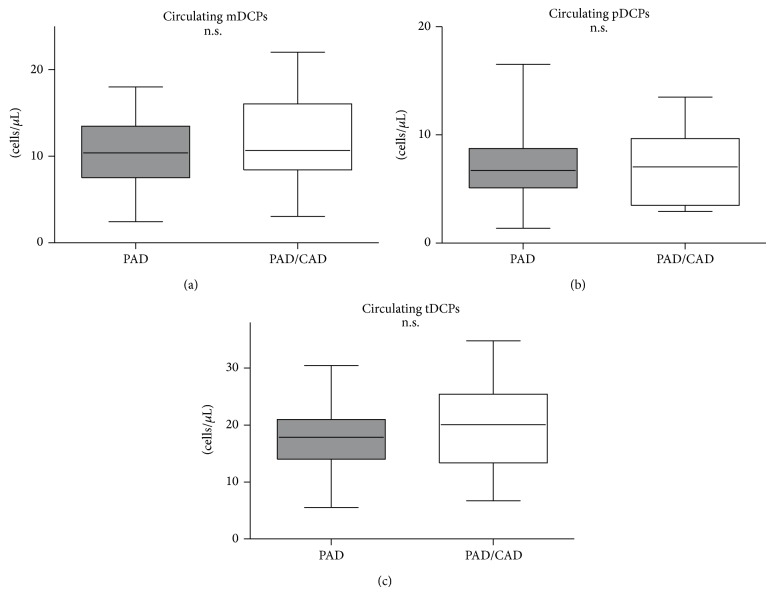
Frequency of circulating mDCPs, pDCPs, and tDCPs in patients with PAD compared with patients with PAD and CAD. Absolute numbers of circulating mDCPs (a), pDCPs (b), and tDCPs (c) (cells/*μ*L) are shown. The box plots indicate the median (line inside the box), 25th and 75th percentile (lower and upper boundary of the box), and 10th and 90th percentile (whiskers outside box).

The 52 patients with known PAD and no evidence of existing CAD were compared with 14 patients with known PAD and CAD. We found comparable numbers of mDCPs (0.15 versus 0.15 and 10.38 versus 10.69 cells/*μ*L; *P* = n.s.), pDCPs (0.09 versus 0.09 and 6.72 versus 7.04 cells/*μ*L; *P* = n.s.), and tDCPs (0.25 versus 0.26 and 17.84 versus 20.07 cells/*μ*L; *P* = n.s.) (relative as well as absolute numbers) in patients with PAD and CAD (Figure 4).

### 3.6. Immunohistochemical Analysis

The clinical characteristics of the PAD patients that underwent femoralis TEA were as follows: median age 73 years; 75% males; 92% arterial hypertension; 33% diabetes mellitus; 33% hyperlipidemia; 17% smoking; median leukocytes 13.8 gpt/L, median CRP 47,2 mg/dL, and median creatinine 68 mmol/L. The median age of the subjects from which we obtained the control tissue was 40 years. As it was known, those patients had no history of any atherosclerotic diseases, but due to the circumstances of death we had no detailed medical information about them.

In the present study, the occurrence of dendritic cells was analyzed in 12 advanced plaques. For analysis of mDCs, the number of CD209+ expressed by immature mDCs and CD83+ expressed by mature DCs was evaluated. Immunostaining with these markers showed a significantly higher cells number of immature as well as mature mDCs in femoralis plaques compared to arteries without signs of atherosclerosis: 16 versus 8 CD209+ mDCs/0.1 mm^2^ (*P* = 0.012) and 19 versus 5 CD83+ mDCs/0.1 mm^2^ (*P* = 0.034).

In immunohistochemical analysis of pDCs characterized by the markers CD304 and CD123, only very low cells numbers were detected in femoral plaques (3 pDCs/0.1 mm^2^ for both markers) of PAD patients with no difference to control arteries (3 CD123+ pDCs/0.1 mm^2^ and 4 CD304+ pDCs/0.1 mm^2^) as it has been formerly described [22].

**Figure 5:**
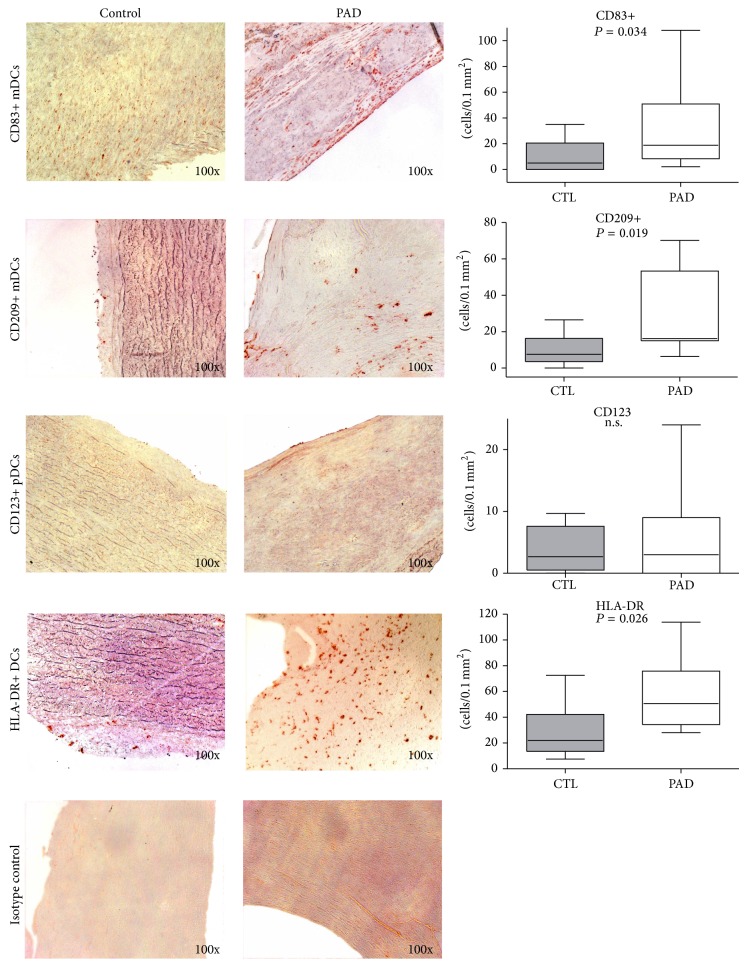
Emergence of myeloid and plasmacytoid DCs as well as antigen-presenting cells in femoralis plaques. CD209+ immature mDCs, CD83+ mature mDCs, CD123+ pDCs, and HLA-DR+ activated APCs in control vessels and femoralis plaques. Also shown: negative (isotype) controls.

Additionally, immunostaining with HLA-DR, a functional marker expressed by activated antigen-presenting cells, revealed a significantly higher cells number in femoral plaques of PAD patients compared to healthy arteries (median 51 versus 22 HLA-DR+ DCs/0.1 mm^2^, *P* = 0.026) (Figure 5).

## 4. Discussion

Peripheral arterial disease (PAD) is a manifestation of systemic atherosclerosis and affects about 10–15% of the general population. PAD is often asymptomatic, leading to underdiagnosis and undertreatment [27]. Symptomatic PAD patients have a worse cardiac prognosis than patients with CAD or cerebrovascular disease [27]. Among the different types of manifestations of atherosclerosis, PAD has the highest 1-year atherothrombotic event rate in comparison to patients with CAD or cerebrovascular disease [9]. In the future, this disease will be even more prevalent since the age of people in the industrialized countries is increasing [28]. An increased inflammatory status is associated with the development of atherosclerosis in the arteries of the lower limbs [29]. Dendritic cells as potent antigen-presenting cells are known to be main players in the process of inflammation.

In the present study, we investigated the number of circulating DCPs in patients with PAD compared to control patients. As a main finding, we were able to demonstrate for the first time that circulating mDCPs, pDCPs, and tDCPs were significantly reduced in patients with PAD. In addition, we showed that these cells can be found in femoral plaques of patients with peripheral atherosclerosis.

In former studies, a significant reduction of circulating DCPs in patients with different manifestations of atherosclerosis was demonstrated [18–21]. Circulating DCPs were found to be reduced in patients with stable and unstable CAD, with AMI, and with stroke [18, 19, 21]. However there is only one report focussing on circulating DCPs in patients with PAD [14]. In contrast to our own findings, they report an increase of the relative numbers of circulating mDCPs and a reduction of relative numbers of circulating pDCPs in patients suffering from PAD. Total DCs count and absolute DCs numbers were not altered in comparison to the control group. This is a surprising finding, because, as PAD is a manifestation of atherosclerotic disease, one could speculate that DCPs in patients with PAD would behave like DCPs in individuals with CAD and/or cerebrovascular disease. One explanation for the different results of this study in PAD patients might be the use of different marker molecules to identify DCs. Whereas Dopheide et al. used CD11c for mDC and CD123 for pDC, we used the Blood Dendritic Cell Enumeration kit (BDCA kit, Miltenyi Biotec) with BDCA-1 for mDCPs and BDCA-2 for pDCPs which might provide more sensitive and reliable results [14]. In our PAD patients group, we found elevated CRP levels (but still within normal range) as marker for higher inflammation. Elevated CRP was found to be an independent marker of increased cardiovascular risk [30, 31] and of major cardiovascular events [32].

In parallel to the reduction of circulating DCPs in the blood, we could detect mDCs and pDCs in femoral plaques. The numbers of anti-CD209 cells indicative for immature mDCs and DC83+ for mature DCs were significantly elevated in comparison to the control vessels. In contrast, only low numbers of CD304+ and CD123+ cells were found in femoral plaques as well as control vessels.

These results led us to the hypothesis that circulating DCPs might be decreased due to their enhanced recruitment in peripheral atheromas. The fact that an enhanced recruitment into peripheral tissues can lead to a reduction of the pool of circulating DCPs has been shown in previous studies. In acute myocardial infarction, we demonstrated that circulating DCPs were significantly reduced and that this phenomenon is accompanied by their enhanced appearance in the infarcted myocardium [21]. This finding is in concordance with former studies [22, 33]. As mechanism for the enhanced recruitment of circulating DCPs into the vascular wall enhanced expression of MIP-3 alpha, a potent and specific chemokine for the attraction of DCs has been shown in carotid plaques in patients with cerebrovascular disease [22].

The low number of pDCs in the plaque tissue is not a surprising finding because studies demonstrate that pDCS were not recruited, or only to a low number, into the inflamed tissue [22]. Yilmaz et al. showed that pDCs were comparable in vulnerable and stable carotid plaques whereas the number of mDCs was significantly elevated in unstable plaques [22]. The result of low pDCs in plaques is in concordance with our finding that in patients with PAD the reduction of circulating mDCPs was much more pronounced than pDCPs. A possible explanation for this result is the fact that pDCPs are the major DC subset found in lymph nodes [34]. Furthermore, we regarded patients with combined manifestations of atherosclerosis. In this context, in patients with CAD and PAD, a comparable reduction of circulating DCPs was observed compared to patients with PAD as single manifestation. This might be an explanation that patients with PAD have an even worse cardiac prognosis than patients with CAD [27].

We could not completely exclude that other reasons such as reduced release from bone marrow and/or differentiation and/or apoptosis might be responsible for the reduction of circulating DCPs in PAD patients which would not be surprising, since PAD can be regarded as chronic inflammatory disease. Some studies were published dealing with cytokine levels in CAD patients which are associated with DC maturation. Equal concentrations of growth factor granulocyte-macrophage colony stimulating factor (GM-CSF) were found in patients with CAD compared to control patients [35]. It has been shown that GM-CSF is a main mediator of DC maturation leading to inflammation in different inflammatory disorders [36] so that it could be possible that this haematopoetic cytokine plays an important role in DC maturation in patients with PAD. If the concentration in PAD patients compared to CAD would be equal, this argues against reduced differentiation of DCPs in PAD patients.

The FMS-like tyrosine kinase 3 ligand (Ft3L) is a key cytokine involved in DC development and release from the bone marrow [37]. A recent study revealed that plasma levels of Ft3L were significantly reduced in CAD patients and positively correlated with DC levels [36]. Therefore, it could not be excluded that in patients with PAD a reduced release of DCPs from the bone marrow could be responsible for our finding of decreased levels in circulating DCPs. Until now, there are no studies dealing with those issues in PAD. As long as these findings were only demonstrated in patients with CAD, we could only speculate about the influence of these cytokines in peripheral atherosclerosis.

## 5. Conclusion

In our present study, we demonstrated for the first time a significant decrease in circulating mDCPs as well as, to lower extent, in pDCPs in patients with PAD. In addition, we found markers indicative for mDCs in atherosclerotic femoral plaques. Future studies are required to define the true association of inflammation and DCPs in PAD.

## Figures and Tables

**Table 1:** 

Antibody	Source	Dilution	Specificity

CD209	BD	1 : 100	Immature DCs
CD83	Serotec	1 : 40	Mature DCs
CD304	Miltenyi Biotec	1 : 100	pDCs
CD123	Serotec	1 : 100	pDCS
HLA-DR	Dako	1 : 15	Activated APCs

DCs: dendritic cells; APCs: antigen-presenting cells.

**Table 2:** 

	PAD	Control	*P* value

*n*	52	60	
Age (years)	67.5	65	n.s.
Male gender (%)	83	45	<0.001
Diabetes mellitus (%)	37	20	n.s.
Hypertension (%)	88	76	n.s.
Smoking (%)	78	47	0.001
Hyperlipidemia (%)	75	52	0.01
CRP (mg/dL)	3.0	2.0	0.02
Leucocytes (Gpt/L)	7.2	7.3	n.s.
Urea (mmol/L)	5.1	5.4	n.s.
Creatinine (*µ*mol/L)	75.5	72	n.s.
Total cholesterol (mmol/L)	4.7	5.4	0.002
LDL cholesterol (mmol/L)	2.6	3.3	<0.0001
HDL cholesterol (mmol/L)	1.2	1.3	n.s.
Triglycerides (mg/dL)	1.7	1.3	0.006

Values are reported as means or percentages. HDL: high-density lipoprotein; LDL: low-density lipoprotein.

## References

[1] Brodie B. C. (1846). *Lectures Illustrative of Various Subjects in Pathology and Surgery*.

[2] Hirsch A. T., Haskal Z. J., Hertzer N. R. (2006). ACC/AHA 2005 guidelines for the management of patients with peripheral arterial disease (lower extremity, renal, mesenteric, and abdominal aortic): executive summary a collaborative report from the American Association for Vascular Surgery/Society for Vascular Surgery, Society for Cardiovascular Angiography and Interventions, Society for Vascular Medicine and Biology, Society of Interventional Radiology, and the ACC/AHA Task Force on Practice Guidelines (Writing Committee to Develop Guidelines for the Management of Patients With Peripheral Arterial Disease) endorsed by the American Association of Cardiovascular and Pulmonary Rehabilitation; National H art, Lung, and Blood Institute; Society for Vascular Nursing; TransAtlantic Inter-Society Consensus; and Vascular Disease Foundation. *Journal of the American College of Cardiology*.

[3] O'Riordain D. S., O'Donnell J. A. (1991). Realistic expectations for the patient with intermittent claudication. *British Journal of Surgery*.

[4] Criqui M. H., Langer R. D., Fronek A. (1992). Mortality over a period of 10 years in patients with peripheral arterial disease. *The New England Journal of Medicine*.

[5] Leng G. C., Lee A. J., Fowkes F. G. R. (1996). Incidence, natural history and cardiovascular events in symptomatic and asymptomatic peripheral arterial disease in the general population. *International Journal of Epidemiology*.

[6] Brevetti G., Martone V. D., Perna S. (1998). Intermittent claudication and risk of cardiovascular events. *Angiology*.

[7] Stammers F. A. (1954). Peripheral arterial disease; some points of common interest to general and orthopaedic surgery. *The Journal of Bone and Joint Surgery. British Volume*.

[8] Allen E. V., Barker N. W., Hines E. A. (1946). *Peripheral Vascular Diseases*.

[9] Steg G., Bhatt D. L., Wilson P. W. F. (2007). One-year cardiovascular event rates in outpatients with atherothrombosis. *Journal of the American Medical Association*.

[10] Osler W. (1908). Diseases of the arteries. *Modern Medicine: Its Practice and Theory*.

[11] Ross R. (1999). Atherosclerosis—an inflammatory disease. *The New England Journal of Medicine*.

[12] Ridker P. M., Cushman M., Stampfer M. J., Tracy R. P., Hennekens C. H. (1998). Plasma concentration of C-reactive protein and risk of developing peripheral vascular disease. *Circulation*.

[13] Tzoulaki I., Murray G. D., Lee A. J., Rumley A., Lowe G. D. O., Fowkes F. G. R. (2007). Inflammatory, haemostatic, and rheological markers for incident peripheral arterial disease: Edinburgh Artery Study. *European Heart Journal*.

[14] Dopheide J. F., Obst V., Doppler C. (2012). Phenotypic characterisation of pro-inflammatory monocytes and dendritic cells in peripheral arterial disease. *Thrombosis and Haemostasis*.

[15] Steinman R. M., Cohn Z. A. (1973). Identification of a novel cell type in peripheral lymphoid organs of mice. I. Morphology, quantitation, tissue distribution. *The Journal of Experimental Medicine*.

[16] Banchereau J., Briere F., Caux C. (2000). Immunobiology of dendritic cells. *Annual Review of Immunology*.

[17] Bobryshev Y. V., Lord R. S. A. (1995). Ultrastructural recognition of cells with dendritic cell morphology in human aortic intima. Contacting interactions of vascular dendritic cells in athero-resistant and athero-prone areas of the normal aorta. *Archives of Histology and Cytology*.

[18] Yilmaz A., Schaller T., Cicha I. (2009). Predictive value of the decrease in circulating dendritic cell precursors in stable coronary artery disease. *Clinical Science*.

[19] van Vré E. A., Hoymans V. Y., Bult H. (2006). Decreased number of circulating plasmacytoid dendritic cells in patients with atherosclerotic coronary artery disease. *Coronary Artery Disease*.

[20] van Brussel I., van Vré E. A., de Meyer G. R. Y., Vrints C. J., Bosmans J. M., Bult H. (2010). Expression of dendritic cell markers CD11c/BDCA-1 and CD123/BDCA-2 in coronary artery disease upon activation in whole blood. *Journal of Immunological Methods*.

[21] Kretzschmar D., Betge S., Windisch A. (2012). Recruitment of circulating dendritic cell precursors into the infarcted myocardium and pro-inflammatory response in acute myocardial infarction. *Clinical Science*.

[22] Yilmaz A., Weber J., Cicha I. (2006). Decrease in circulating myeloid dendritic cell precursors in coronary artery disease. *Journal of the American College of Cardiology*.

[23] Norgren L., Hiatt W. R., Dormandy J. A., Nehler M. R., Harris K. A., Fowkes F. G. R. (2007). Inter-society consensus for the management of peripheral arterial disease (TASC II). *European Journal of Vascular and Endovascular Surgery*.

[24] Paul K., Kretzschmar D., Yilmaz A. (2013). Circulating dendritic cell precursors in chronic kidney disease: a cross-sectional study. *BMC Nephrology*.

[25] Stary H. C., Chandler A. B., Dinsmore R. E. (1995). A definition of advanced types of atherosclerotic lesions and a histological classification of atherosclerosis: a report from the Committee on Vascular Lesions of the Council on Arteriosclerosis, American Heart Association. *Arteriosclerosis, Thrombosis, and Vascular Biology*.

[26] Yilmaz A., Lochno M., Traeg F. (2004). Emergence of dendritic cells in rupture-prone regions of vulnerable carotid plaques. *Atherosclerosis*.

[27] Au T. B., Golledge J., Walker P. J., Haigh K., Nelson M. (2013). Peripheral arterial disease: diagnosis and management in general practice. *Australian Family Physician*.

[28] McDermott M. M. (2013). Functional impairment in peripheral artery disease and how to improve it in 2013. *Current Cardiology Reports*.

[29] Brevetti G., Schiano V., Verdoliva S. (2006). Peripheral arterial disease and cardiovascular risk in Italy. Results of the Peripheral Arteriopathy and Cardiovascular Events (PACE) study. *Journal of Cardiovascular Medicine*.

[30] Pearson T. A., Mensah G. A., Alexander R. W. (2003). Markers of inflammation and cardiovascular disease: application to clinical and public health practice: a statement for healthcare professionals from the centers for disease control and prevention and the American Heart Association. *Circulation*.

[31] Beckman J. A., Preis O., Ridker P. M., Gerhard-Herman M. (2005). Comparison of usefulness of inflammatory markers in patients with versus without peripheral arterial disease in predicting adverse cardiovascular outcomes (myocardial infarction, stroke, and death). *The American Journal of Cardiology*.

[32] Schlager O., Amighi J., Haumer M. (2009). Inflammation and adverse cardiovascular outcome in patients with renal artery stenosis and peripheral artery disease. *Atherosclerosis*.

[33] Yilmaz A., Dietel B., Cicha I. (2010). Emergence of dendritic cells in the myocardium after acute myocardial infarction—implications for inflammatory myocardial damage. *International Journal of Biomedical Science*.

[34] Lore K., Darzynkiewicz Z., Roederer M., Tanke H. J. (2004). Isolation and immunophenotyping of human and rhesus macaque dendritic cells. *Methods in Cytometry: New Developments*.

[35] van Brussel I., van Vré E. A., De Meyer G. R. Y., Vrints C. J., Bosmans J. M., Bult H. (2011). Decreased numbers of peripheral blood dendritic cells in patients with coronary artery disease are associated with diminished plasma Flt3 ligand levels and impaired plasmacytoid dendritic cell function. *Clinical Science*.

[36] Hamilton J. A. (2002). GM-CSF in inflammation and autoimmunity. *Trends in Immunology*.

[37] Kingston D., Schmid M. A., Onai N., Obata-Onai A., Baumjohann D., Manz M. G. (2009). The concerted action of GM-CSF and Flt3-ligand on in vivo dendritic cell homeostasis. *Blood*.

